# Efficacy and Safety of Pomegranate Medicinal Products for Cancer

**DOI:** 10.1155/2015/258598

**Published:** 2015-03-01

**Authors:** Christian Vlachojannis, Benno F. Zimmermann, Sigrun Chrubasik-Hausmann

**Affiliations:** ^1^Institute of Forensic Medicine, University of Freiburg, Albertstr. 9, 79104 Freiburg, Germany; ^2^Institute of Nutrition and Food Sciences, Food Technology and Food Biotechnology, University of Bonn, Römerstraße 164, 53117 Bonn, Germany; ^3^Institut Prof. Dr. Georg Kurz GmbH, Eupener Straße 161, 50933 Köln, Germany

## Abstract

Preclinical *in vitro* and *in vivo* studies demonstrate potent effects of pomegranate preparations in cancer cell lines and animal models with chemically induced cancers. We have carried out one systematic review of the effectiveness of pomegranate products in the treatment of cancer and another on their safety. The PubMed search provided 162 references for pomegranate and cancer and 122 references for pomegranate and safety/toxicity. We identified 4 clinical studies investigating 3 pomegranate products, of which one was inappropriate because of the low polyphenol content. The evidence of clinical effectiveness was poor because the quality of the studies was poor. Although there is no concern over safety with the doses used in the clinical studies, pomegranate preparations may be harmful by inducing synthetic drug metabolism through activation of liver enzymes. We have analysed various pomegranate products for their content of anthocyanins, punicalagin, and ellagic acid in order to compare them with the benchmark doses from published data. If the amount of coactive constituents is not declared, patients risk not benefiting from the putative pomegranate effects. Moreover, pomegranate end products are affected by many determinants. Their declaration should be incorporated into the regulatory guidance and controlled before pomegranate products enter the market.

## 1. Introduction

Pomegranate products are amongst most promising antitumorigenic dietary supplements. The polyphenol fraction of pomegranate exerts antiproliferative and proapoptotic effects in a number of cancer cell lines [1]. Various mediators of carcinogenesis are inhibited by the pomegranate active principle* in vitro*, for example, vascular endothelial growth factor [2], insulin-like growth factors [3], cytokine-stimulated NF-*κ*B [4], and others [5, 6]. Fermentation is a new technology that enriches coactive compounds [7]. Fermentation of pomegranate juice with* Lactobacillus plantarum *increased the concentration of ellagic acid and enhanced the antimicrobial activity of the juice. Both fresh and fermented juices inhibited the growth of K562 tumor cells [7]. Polyphenols from fermented pomegranate juice showed about twice the antiproliferative effect shown by polyphenols from fresh pomegranate juice. They also inhibited the activity of aromatase and 17-*β*-hydroxysteroid dehydrogenase type 1 by 60–80% and inhibited chemically induced formation of cancerous lesions in a murine mammary gland organ culture by about 50% [8] However, a specific purified polyphenol compound and pomegranate seed oil were more effective than fermented juice in this* in vitro* test [9]. The isolated ellagitannins, punicalagin, and ellagic acid also had a high antiproliferative activity against various cancer cell lines [10, 11].

The aromatase enzyme, which converts androgen to estrogen, plays a key role in breast cancer. Tamoxifen is the usual antiestrogen therapy for hormone-receptor-positive breast cancer in premenopausal women, though it carries a risk of development of resistance [12]. Pomegranate fruit extracts enhanced the action of tamoxifen in both tamoxifen-sensitive and tamoxifenresistant breast cancer cells, through the inhibition of cell viability by inducing the cell-death machinery [13].

The polyphenols also inhibited the expression of genes for key androgen-synthesizing enzymes and androgen receptors, suggesting that the pomegranate polyphenols (mainly the oligomeric punicalagin and the monomeric ellagic acid) affect androgen-independent prostate cancer cells and the subset of human prostate cancer cells where the androgen receptor is upregulated [4, 14]. In investigations of the anti-invasive effects of ellagic acid in androgen-independent human and rat prostate cancer cell lines* in vitro*, ellagic acid significantly inhibited the motility and invasion of cells examined in migration and invasion assays. The secretion of matrix metalloproteinases from androgen-independent human and rat prostate cancer cell lines and the proteolytic activity of collagenase/gelatinase were significantly reduced, indicating that the anti-invasive potential of prostate cancer cells is mediated via protease activity [15].

Although all pomegranate-derived materials contribute to a greater or lesser extent to the anticancer effect of pomegranate, the polyphenol fraction and supercritical CO_2_-extracted seed oil were more potent than cold-pressed pomegranate seed oil in inhibiting growth of prostate cancer xenografts in athymic mice [16]. Pomegranate juice was more effective than compounds isolated from the juice [17].

In immunodeficient mice, pomegranate juice and extract inhibited tumor-associated angiogenesis and slowed the growth of prostate cancer [18]. Oral infusion of pomegranate fruit extract resulted not only in a dose-dependent inhibition of tumor growth, but also in a decrease of prostate-specific antigen (PSA) levels in mice implanted with androgen-responsive cancer cells [19]. Tumor reductions were also seen in mice with induced lung, colon, and skin cancers [1].

Of the polyphenols, the chemopreventive pomegranate ellagitannins (e.g., punicalagin and punicalin) are metabolized during absorption. One of the metabolites, ellagic acid, is further metabolized by the colonic microflora to urolithin A. Both ellagic acid and urolithin A contribute to the mechanism of anticancer action, but urolithin was less effective in inhibiting cancer cell proliferation [20–22]. There was no difference in metabolite production between pomegranate juice and extracts thereof [23], though inactive ellagitannin-derived metabolites are also produced by the colonic microflora [24]. This may account for individual differences in the response to pomegranate consumption.

The aim of this study was to summarize data on the clinical effectiveness and safety of pomegranate preparations for the treatment of cancer and to analyse various pomegranate preparations for their content of coactive compounds in order to find out the dose required for an anticancer effect in patients suffering from prostate cancer.

## 2. Methods

### 2.1. Systematic Reviews on Effectiveness and Safety

On June 20, 2014 we searched PubMed using the terms: “pomegranate cancer” and “pomegranate prostate” and, on June 29, “pomegranate safety” and “pomegranate toxicity,” the reference lists of articles were searched by hand for other publications. No methodological filter was applied and the search was not limited by language. The full manuscript was retrieved for each record that had a chance of meeting the review criteria (clinical trial, safety investigation). Two authors (Christian Vlachojannis and Sigrun Chrubasik-Hausmann) extracted the data independently and evaluated the quality of the studies and the strength of the evidence of clinical effectiveness using the same criteria as in previous reviews [25–35]. Briefly, the assessment of quality was based on “yes” or “no” answers to the following questions: was or were

- patients included on the basis of specified eligibility criteria;
- randomization appropriate;
- treatment allocation concealed;
- baseline values of the groups similar
- outcome measures and control interventions explicitly described;
- cointerventions comparable;
- outcome measures relevant;
- adverse events fully described;
- attrition of patients from the study (the “drop-outs”) fully described;
- sample size based on a priori power calculation;
- analysis by intention-to-treat in the event of attrition of patients during the study;
- point estimates and measures of variability presented for the primary outcome measure;
- studies undertaken over an appropriate time-course to demonstrate the putative effect.

For observational studies, some of the questions are not applicable but the inability to supply a “yes” answer itself marks an “absence of quality” in systematic reviews of this sort. Potential disagreements were discussed and resolved by referring to the original protocol.

Adding up the “yes” answers applicable to each study gave it a total score (TS) out of a maximum of 13. Evidence of effectiveness was defined as (i) “strong”: pooling of data from at least 2 confirmatory studies demonstrating a clinically relevant effect;

(ii) “moderate”: consistent findings from one confirmatory study with a clinically relevant effect, multiple exploratory studies of high internal validity (TS 10 and higher), or both;

(iii) poor: multiple exploratory studies of low internal validity or one single study of high internal validity.

### 2.2. Analyses of Various Pomegranate Products

The pomegranate preparations we investigated included

- three commercially available pure (100%) juices:
  - 5174-13, expiry date June 2, 2015,
  - L3074, expiry date Sept 15, 2014,
  - POM Wonderful (expiry date April 21, 2014);
- two juice concentrates:
  (iv) POM Wonderful expiry date August 1, 2014
  (v) F4, a commercially available fermented pomegranate concentrate supplemented with 10% elderberry concentrate (details not stated; photometric assessment on May 30, 2013);
- five extracts:
  (vi) POMx capsules (1000 mg capsules, expiry date June 17, 2015),
  (vii) ultra Granatapfel forte capsules (500 mg, expiry date Sept., 2015),
  (viii) extract 20651 (not commercially available, batch 19829, native drug extract ratio 5.3–8 : 1, solvent ethanol),
  (ix) GranaProstan capsules (500 mg freeze-dried powder from fermented pomegranate juice (84%),
  (x) pomegranate extract (16%, drug extract ratio and solvent not stated, photometric assessment in February 2013, expiry date Feb 18, 2015).

Punicalagins A and B and ellagic acid were analysed by RP-UHPLC-UV using authentic reference compounds. Anthocyanidins were analysed by RP-UHPLC-Vis using cyanidin-3-O-glycoside as reference. Total polyphenols were determined by the Folin-Ciocalteu photometric method using gallic acid as reference. Details of the methods are presented at http://www.uniklinik-freiburg.de/rechtsmedizin/forschung/phytomedizin.html.

## 3. Results

### 3.1. Systematic Reviews on Effectiveness and Safety

**Figure 1:**
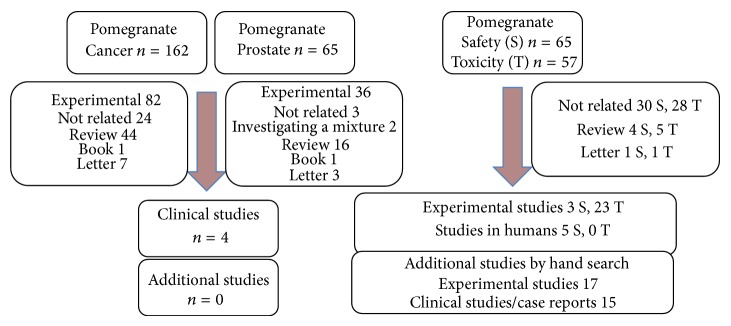
Search profile.

We identified 162 references for “pomegranate cancer” and 65 references for “pomegranate prostate,” both included 4 clinical studies investigating pomegranate products in prostate cancer patients (Figure 1, see webpage: PubMed searches). The quality of the studies is listed in Table 1. According to the criteria set out above in the methods, the evidence of effectiveness of pomegranate products for the treatment of prostate cancer is poor.

We identified 42 references for “pomegranate safety” and 57 for “pomegranate toxicity,” respectively. A total of 26 experimental and 5 clinical studies were included in the part on safety together with 17 experimental studies and 15 clinical studies from hand searches (Figure 1, see webpage: PubMed searches).

### 3.2. Analyses of Various Pomegranate Products

Table 2 summarizes the total polyphenol content by photometric assessment (Folin-Ciocalteu method) as declared by the manufacturers, along with our own Folin-Ciocalteu data. The HPLC chromatograms are placed on the above-mentioned webpage (see Results). The table also summarizes coactive compounds as assessed by HPLC and their sum in mg/L or mg/kg and the daily dose of polyphenols in the doses of product recommended by the manufacturers. It can be seen that the sum of our HPLC measurements of anthocyanins, punicalagins, and ellagic acid is substantially less than the photometrically measured total polyphenols, though there is a correlation of sorts.

The lower part of the table shows the content on coactive compounds in commercially available pomegranate preparations, as taken from the references stated and the calculated sum of polyphenols in the recommended daily doses.

Table 3 lists the individual anthocyanidins measured by HPLC, which also allows a distinction to be made between pomegranate and elderberry anthocyanidins in the juice concentrate F4.

## 4. Discussion

### 4.1. Evidence of Effectiveness of Pomegranate Products

Pomegranate preparations have so far been investigated only in patients with prostate cancer. In an uncontrolled study, patients with rising PSA after surgery or radiation for prostate cancer were treated with 240 mL of fermented pomegranate juice per day, containing total polyphenols equivalent to 570 mg of gallic acid [36]. The content of coactive compounds as assessed by HPLC was not stated but was said in another study [37] to be similar to that in extract POMx, which contained 370 mg punicalagin and 30 mg ellagic acid in the daily dosage [14]. Mean PSA doubling time increased with treatment from a mean of 15 months at baseline to 54 months after treatment [36]. The remaining observational study included 104 men with rising PSA but without metastases. Daily doses of either 1000 or 3000 mg of a polyphenol extract of pomegranate were given (POMx, 37% punicalin (POM Wonderful, LLC; Los Angeles, California, http://cms.herbalgram.org/herbclip/474/051321-474.html. Patients were stratified according to their baseline PSA doubling time and Gleason score. The primary endpoint was the increase in PSA doubling time after 6 months. The average PSA doubling time did indeed increase from 12 months to almost 19 months, irrespective of dose. This may or may not indicate a ceiling effect. The data are not conclusive because of the lack of a placebo and the unreliability of the endpoint [37]. The coactive compound urolithin A was detected more often in benign and malignant prostate tissue in patients who had received POMx during the 4 weeks before surgery. An inverse correlation was expected between intraprostatic urolithin A and the oxidative stress tissue marker 8-hydroxy-2′-deoxyguanosine content. The study was powered to detect a 35% reduction in that marker. However, POMx was associated only with 16% lowermarker content, which was not statistically significant in this short-term clinical trial [38]. It may well be that the 4-week treatment duration was too short. The results are eagerly awaited of two on-going and two as yet unpublished investigations of pomegranate in prostate cancer patients with a juice, a proprietary extract (2 studies) and a liquid extract [39]. Stenner-Liewen et al. [40] carried out a phase IIb, double-blinded, randomized placebo-controlled trial in patients with histologically confirmed prostate cancer in patients with a PSA ≥ 5 ng/mL; this used an amount of pomegranate active principle per day (20 mg in 500 mL), which was only 5% of that investigated in the other studies [41]. Unsurprisingly, the study concluded that daily pomegranate intake has no impact on PSA levels in patients with advanced prostate cancer.

In our critique of the Cochrane reviews on herbal medicines [42], we called for rigorous declaration of coactive ingredients in study medications to avoid misleading interpretations of data. For example, Stenner-Liewen and coworkers [40] relied on photometric assessments of the coactive principle in their study medication, failing to take into account the fact that photometric assessments overestimate the true polyphenol content by detecting all polyphenolic or antioxidative compounds regardless of their clinical activity [41]. The photometric assessment of the total mixed polyphenols in 500 mL of the proprietary pomegranate blend was 1147 mg of gallic acid equivalents. Subtracting the various polyphenols from other components of the blend (white tea and chokeberry—agave concentrate does not contain polyphenols) amounted to 445 mg/500 mL; the remaining value of around 700 mg of pomegranate polyphenols does not reflect the dose of coactive compounds (e.g., punicalagin and ellagic acid), our HPLC analysis resulted in a total of 20 mg. Bench-mark doses of coactive ingredients are given in the study by Paller and coworkers [37], a total of 400 mg per day as assessed by HPLC. Thus the conclusion of the Stenner-Liewen group that daily pomegranate intake has no impact on PSA levels in patients with advanced prostate cancer is wrong since it was based on an inadequate amount of coactive ingredients in their pomegranate mixture. Likewise, similar confusion exists for cranberry products in which the photometric assessments do not reflect the true content of coactive ingredients [43]. HPLC assessments provided bench-mark doses for the prevention of urinary tract infections [44].

Recently, a significant decrease in PSA levels during treatment with pomegranate extract Pomella (225 mg/kg, Table 2) has been demonstrated in a mouse model of prostate cancer. The production of testosterone, DHT, DHEA, androstenedione, androsterone, and pregnenolone was inhibited in prostate cancer cell lines and serum steroids reduced after 20 weeks of treatment (0.17 g/L in drinking water) [45]. In metastatic castration-resistant PCa cells, POMx exhibited potent* in vitro* cytotoxicity and in athymic nude mice, the extract retarded C4-2 tumor growth in skeleton and significantly enhanced the efficacy of docetaxel [46]. These studies and the experiments mentioned in the Introduction of our manuscript suggest that the clinical effectiveness of pomegranate products in the treatment of prostate and other cancers deserves further evaluation.

## 5. Safety Aspects

### 5.1. Based on Experiments

A diet containing 6% punicalagin given to rats for 37 days caused no obvious toxicity [47]. The oral LD_50_ of a pomegranate extract standardized to 30% punicalagins, 5% ellagic acid, and 0.3% gallic acid (photometric assessment 70% polyphenols, trade name POMELLA) was found to be greater than 5 g/kg body weight in rats and mice. The respective* intraperitoneal *LD_50_s in rats and mice were determined as 217 and 187 mg/kg body weight. In a subchronic study in rats, a diet containing up to 600 mg/kg body weight/day of this extract was given over 90 days with or without a 28-day recovery phase. Compared with the control group, giving the extract did not result in any clinically relevant treatment-related organ changes. The “no observed-adverse-effect level” was defined as 600 mg/kg body weight/day, the highest extract dose tested [48].

Pomegranate fruit extract exerted an embryoprotective effect against adriamycin-induced oxidative stress in 12-day old chick embryos. After 24 and 48 h of incubation, 70 *μ*g/egg of adriamycin on its own produced a significant dose versus time-dependent reduction in body weight and volume of amniotic fluid and a dose-related increase in gross embryological deformities and significant changes in the levels of biochemical markers in amniotic fluid. These changes were significantly reduced by preadministration of pomegranate fruit extract at a dose of 200 *μ*g/egg [49]. Lead acetate administration inhibited spermatogenesis in rats by reducing the length of the stages related to spermiation and onset of mitosis. The epididymal sperm number and daily sperm production were reduced. Giving ethanolic pomegranate extract along with the lead acetate resulted in longer spermiation stages than with the lead acetate only. The deleterious effects on epididymal sperm number and daily sperm production were reduced. Thus, pomegranate may prevent lead acetate-induced spermatogenic disruption in rats possibly owing to antioxidant effects [50]. Pomegranate also reduced the RNA-damaging effect of doxorubicin, H_2_O_2_, and spermine. Its inhibitory activity could be related to its ability to form complexes with doxorubicin and H_2_O_2_ or its interaction with the intracellular formation of reactive substances that mediated their toxicity [51]. In adult Wistar rats, pomegranate juice augmented the antioxidant defence mechanism against carbon tetrachloride-induced reproductive toxicity [52]. In other tests, pomegranate extract was found to be protective against methotrexate-induced oxidative bone marrow damage [53], reduced methotrexate-induced neurotoxicity [54], and reversed methotrexate-induced oxidative stress and apoptosis in hepatocytes by modulating Nrf2-NF-*κ*B pathways in male Swiss albino rats. Preparations of pomegranate may, thus, help to reduce some adverse effects of methotrexate. Further tests demonstrated that pomegranate methanolic peel extract inhibited aluminum-induced hepatorenal toxicity [55], mercuric chloride-induced oxidant toxicity [56] and gentamicin-induced nephrotoxicity [57]. Pomegranate seed oil in doses up to 0.64 mg/kg, one hour before 100 mg/kg of the nephrotoxic agent diazinon had a nephroprotective effect [58]. This has been confirmed with hexachlorobutadiene as the nephrotoxic agent [59]. The “no observable adverse effect level” (NOAEL) of pomegranate seed oil was 50.000 ppm PSO (=4.3 g PSO/kg body weight/day) [60].

In hepatitis induced in rats by D-galactosamine/lipopolysaccharide, a 2-week pretreatment with pomegranate juice 20 mL/kg body weight per day protected against hepatic damage by suppressing oxidative stress. Histopathology showed that the pomegranate juice restored the hepatic architecture to normal [61]. Histopathological studies of the liver of rats fed pomegranate fruit extract and carbon tetrachloride also indicated a hepatoprotective effect. Likewise, pomegranate juice protected against carbon tetrachloride-induced hepatotoxicity [62] and nephrotoxicity [63] and protected against ethylene glycol-induced crystal deposition in renal tubules [64] and the development of azoxymethane-induced aberrant crypt foci [65]. Oral pomegranate extract had a protective effect against cisplatin ototoxicity in rats. Cisplatin ototoxicity was assessed by analysing “distortion product otoacoustic emissions” 3 days before and after the cisplatin injections. Histological changes in the cochleas were observed by light microscopy [66]. This was confirmed in an experimental study with aminoglycoside as the ototoxic agent [67] A whole fruit extract of pomegranate was cardioprotective against doxorubicin-induced toxicity [68].

In the chick embryo model, doses of whole fruit extract (DER 3 : 1, solvent ethanol 50%) of less than 0.1 mg per embryo were not toxic. The LD_50_ of the extract, determined after intraperitoneal administration in mice, was 731 mg/kg (confidence limits 565–945 mg/kg). At the doses of 0.4 and 1.2 mg/kg of extract, repeated intranasal administration to Wistar rats produced no toxic effects in terms of food intake, weight gain, behavioural or biochemical measurables, nor was it associated with histopathological changes [69]. Aqueous and lipophilic pomegranate peel extracts have demonstrated a dose-dependent antimutagenic activity in* Salmonella typhimurium* strains [70]; this was probably attributable to the content on ellagitannins [71]. No toxic effects were observed in mice treated with aqueous pomegranate fruit extracts [72]. A study in Swiss mice treated with ethanolic extracts of pomegranate leaf or fruit confirmed the absence of mutagenic effects and the dose-dependent protective effects against cyclophosphamide-induced oxidative DNA damage [73]. However, a later study was carried out on the genotoxicity of whole pomegranate fruit extract (solvent 50% ethanol) using different* in vitro* and* in vivo* assays to detect DNA damage at different expression levels: it indicated that this extract can induce genetic damage at different expression levels: recombinogenic, mutagenic, and clastogenic [74]. Thus, the use of this extract may well carry a genetic risk and an analysis of the balance of risk and benefit is probably crucial. Whereas pomegranate bark [75] and root [76] contain toxic alkaloids, the presence of alkaloids in peel was considered equivocal [77]. Studies of cytotoxicity affecting the Caco-2 cell line and human peripheral blood mononuclear cells (PBMC) could provide preliminary information about toxicity on intestinal cancer cells and normal cells. The effective dose of pomegranate peel extract for stimulating proliferation in Caco-2 cells was 4.7 *μ*g/mL and for PBMCs 44.4 *μ*g/mL [78]. One should therefore be cautious about using peel extract in humans as a natural dietary antioxidant or a therapy (http://archive.lib.cmu.ac.th/full/T/2008/pha0808st_ch4.pdf). However, one should also note that the toxic effects of pomegranate fruit extract occurred at higher doses than the doses used either those in animal experiments or in Cuban folk medicine [69].

### 5.2. Based on Data from Humans

In the clinical study investigating a pomegranate extract in doses of 1000 and 3000 mg, diarrhea occurred more often in the high dose group [37]. Heber et al. [79] carried out two clinical pilot studies on the safety of a pomegranate ellagitannin-enriched polyphenol extract. Sixty-four overweight individuals took one, two, or three 710 mg capsules per day of pomegranate extract for 28 days, each capsule containing 435 mg of gallic acid equivalents (GAEs). In none of the subjects were there any serious adverse events on complete blood count, blood chemistry, and urinalysis. In another 22 overweight subjects, levels of thiobarbituric acid reactive substances (TBARS) were significantly less after receiving 1000 mg pomegranate extract (610 mg of GAEs) versus baseline measurements. Diabetic indicators were not worsened in diabetic patients taking pomegranate juice; serum lipid peroxidases were reduced by 56% and TBSARs by 24% whereas serum SH groups increased by 12% and paraoxonase activity by 24% [80]. In other clinical studies, consumption of pomegranate juice or extracts were also well tolerated [36, 38, 40, 81–85]. No toxic effects were seen in a one-year pilot study of the proprietary pomegranate extract POMx in 10 patients with carotid artery stenosis (5 of whom continued taking the extract another 2 years) [86]. Interestingly, the improvement in clinical signs took place during the first 12 months of the study but was maintained over the following 2 years. Pomegranate fruit and peel extracts have so far been used safely from a toxicological perspective [87].

Allergies to pomegranate may occur but are very rare [88–92]. One case report described exercise-induced anaphylaxis triggered by the ingestion of pomegranate, the allergy being confirmed by immunoblotting and absence of lipid transfer protein cross-reactivity, although exercise-induced anaphylaxis is generally independent of the kind of food ingested before exercise [93]. Mannitol which is also contained in pomegranate has been identified as causing IgE-mediated hypersensitivity [94].

### 5.3. Risk of Interactions

If pomegranate preparations are taken over longer periods, putative interactions with other medications need to be considered. This is because the pomegranate active principle interacts with hepatic cytochrome P450 [95, 96]. The* in vitro* 1′-hydroxylase activity of midazolam, catalysed by human CYP3A, was inhibited less by a commercial pomegranate juice than by the juices from grapefruit, black mulberry, and wild grape [97]. Pomegranate juice did not impair the clearance of oral or intravenous midazolam in volunteers, [98]. However, rhabdomyolysis has been associated with pomegranate juice consumption in a patient taking synthetic rosuvastatin, though the latter is not known to be metabolized by hepatic P450 3A4 [99]. More studies are needed to determine whether these and other interactions such as the interaction between pomegranate-containing products and the immunosuppressive agent tacrolimus [100] are clinically significant [101].

### 5.4. Analyses of Various Pomegranate Products

The review of the literature indicates that the active principle of pomegranate may well have a potent anticancer potential, but the clinical evidence of effectiveness is still poor because of the poor quality of the available clinical studies. (The results of four further studies are awaited.) Hong and coworkers described the POMx extract as containing monomeric and oligomeric ellagitannins (punicalagin 37–40% and 3.4% free ellagic acid) but no anthocyanins as determined by high performance-liquid chromatography. Thus, a dose of 1000 mg of extract contained 400 mg of both ellagitannins. Paller et al. [37] stated that each POMx capsule contained 1000 mg of polyphenol extract, comparable to about 8 oz (about 240 mL) of pomegranate juice. According to the “POM wonderful pomegranate juice monograph” of the American Botanical Council [102], the juice contained 1.74 mg/mL punicalagin and 0.14 mg/mL ellagic acid, a dose of 94 mg all together in 240 mL. According to the voice message from the company (see webpage: voice message) one POMx pill contained 370 mg punicalagin. This dose has also been mentioned in the review by Kroeger et al. [39]. But according to our measurements, POMx capsules contained only 132 mg of punicalagin and ellagic acid (combined). Since 3000 mg have not been more effective than 1000 mg, the optimum dose until a ceiling effect occurs may be in between these doses (1000 and 3000 mg) or the ceiling effect may even occur at a dose* less than* 1000 mg. This needs to be clarified in a careful dose-finding study.

A competing company has developed their extract POMELLA based on work at the University of California, Los Angeles. The extract (drug : extract ratio: 50 : 1, solvent not stated) is standardized by HPLC on 30% of punicalagins in addition to smaller amounts of other marker compounds that exist at concentrations less than 5% (ellagic acid, gallic acid, and gallagic acid). Batch (Lot number LPR1EP1212L09) contained 300 mg/1000 mg punicalagin and 20 mg/ellagic acid/1000 mg (see http://pomextract.com/Pomella-Story_fc7cfcf6fd873a1634.html). A daily dose of 1000 mg of this extract contains at least 320 mg of total polyphenols and is presently being tested in a clinical study [39]. The photometrically assessed polyphenol content varies between 60 and 70% ([48]; see webpage POMELLA)

For colorimetric quantification of polyphenolic antioxidants in general, the Folin-Ciocalteu assay is used with gallic acid as reference [103]. Theoretically, however, any polyphenol could be used as reference compound (e.g., pyrogallol (Table 1)). Martin et al. [104] proposed replacing gallic acid by a purified pomegranate pomace extract in the Folin-Ciocalteu assay. This purified extract contained at least 5.6% nonpolyphenols (identified as sugars, moisture, ash, and nitrogen (Kjeldhal assay)) compared with 9.1% in the raw POMx extract (trademark) [104]. The polyphenolic composition of this purified extract has not been quantified in terms of pure reference compounds. Data are not presented as absolute values but as rough estimates of polyphenol contents expressed as percentages of total polyphenols. When POMx extract was analyzed by Folin-Ciocalteu using the purified extract as reference, the result (unsurprisingly) was 92.6%. This percentage does not necessarily reflect 92.6% of polyphenols, since the absolute polyphenolic content of the purified pomegranate pomace extract has not been analysed. Thus, though the purified pomegranate pomace extract may well be appropriate for quality control of the POMx extraction process, it does not allow quantification of polyphenols in pomegranate products as suggested by Martin et al. [104]. The 15.7% of punicalagin expressed as a percentage of the 92.6% total polyphenols suggests a putative absolute value of 14.5% of punicalagin in POMx. It remains questionable why POMx has been characterized as extract standardized on 37–40% punicalagin assessed by HPLC [14]. The Folin-Ciocalteu assay has never claimed to reflect the absolute polyphenol content of a sample. It has been designed as an index for comparing similar samples [105] by using gallic acid as reference compound. The Folin-Ciocalteu values were not declared on the POMx capsules we bought.

Pomegranate preparations can contain up to 48 phenolic compounds, and the complexity of their polyphenolic profiles necessitates the use of hyphenated techniques for a thorough evaluation of their composition [106–108]. For reasons of expense, only punicalagin, ellagic acid, and anthocyanins are measured in routine laboratories despite the presence of larger concentrations of other ellagitannins in processed pomegranate preparations, as shown by Fischer et al. 2011 [107, 108]. Although, if all polyphenols in pure pomegranate preparations were included in the HPLC analysis, resulting estimates of total polyphenol content correlated well with photometric estimates, the correlations were very poor if only punicalagin, ellagic acid, and anthocyanins were used. Our results substantiate this (Table 2).

Standardization of products solely on photometric assessments can be misleading and the content of punicalagin, ellagic acid, and anthocyanins as assessed by routine HPLC should be declared on product labels in addition to the photometric estimates. Both indicators should replace descriptions such as the one that came with the extract “Ultra Granatapfel forte,” claiming that “the punicalagin dose in one capsule is equivalent to 840 mL mother juice” (see webpage: Ultra Granatapfel forte Capsules). Such information is unhelpful because one 500 mg capsule contained only 20 mg polyphenols as assessed by HPLC. Depending on what is taken as the benchmark daily dose for prostate cancer—130 or 400 mg ellagitannins—, many capsules of this product may need to be taken daily, which would be inconvenient as well as expensive.

The dose of oral pomegranate fruit extract chosen in mice to inhibit tumorigenesis was based on the assumption that a typical healthy 70 kg individual may be persuaded to drink 500 mL of pomegranate juice extracted from two fruits [109], containing a putative polyphenol dose of around 350 mg per day (Table 2). Of the products investigated, this dose is contained in 1000 mg of POMx extract if we can rely on the study by Hong et al. [14] or 3 POMx capsules as currently available (Table 2), in 350 mL (3.5 cups) of pure juice L3074, in 12 mL of the concentrated fermented juice F4 supplemented with elderberry concentrate, in 2-3 of the proprietary capsules GranaProstan, or in 16 of the proprietary capsules Ultra Granatapfel forte (an inappropriate dose). Though these doses are large, they can be used safely in patients [110].

Tables 2 and 3 show that the quantity and the spectrum of phenolic compounds vary greatly in different products, depending partly on the ripeness of the fruits [111]. Fresh pomegranates contained between 11 and 1543 mg anthocyanins/L depending on the colour of the variety, white, rose, dark red, and purple [112]. Fresh juices contained 904 to 2067 mg/L of total phenols as assessed by Folin-Ciocalteu [112]. This is in accordance with the study by Gómez-Caravaca and coworkers [113] who found that the total phenolic content ranged from 581 to 2551 mg/L in the pomegranate juices they investigated. Table 2 shows that only one of our pure juices was within this range. Our anthocyanin: polyphenol ratios were lower than those reported by Gómez-Caravaca and coworkers [113], which varied between 20 to 82%; this may well indicate anthocyanin degradation in the samples we analysed.

Reductions or losses of phenolic compounds have been reported in commercial juices, and these have been attributed to commercial processing procedures [114]. Although mother juices (100% pure juices) should contain more polyphenols than blended juices, only 3 of 6 pure juices were rich in ellagitannins and antioxidant capacity. Only one of the 6 pure juices that were rich in ellagitannin was also rich in anthocyanins. Some of the other pure juices had even a lower antioxidant capacity than blended juices. In some juices the antioxidant capacity was attributable to vitamin C rather than to phenolic compounds [106]. Vitamin C may preserve coactive compounds [115].

Factors affecting the stability of anthocyanins in juices include pH, the presence of enzymes and copigments such as metallic ions and sugars and, such processing features as the intensity and duration of heating, the storage temperature, and time and the presence of oxygen and/or light. Short-term thermal treatments (65 and 90°C for 30 or 5 s) decreased the percentage of polymeric anthocyanins and increased the amount of monomeric anthocyanins and thus the bioavailability of coactive compounds [116]. Pasteurization had no influence on the total polyphenols and antioxidant capacity of juices. However, the storage temperature was the main factor affecting all coactive compounds, the total monomeric and individual anthocyanins, the total phenolic compounds, and therefore also the antioxidant activity [116]. Fast degradation of anthocyanins was observed in juices stored at 25°C, while refrigerated storage at 5°C resulted in much slower degradation. Cyanidin-3-O-glucoside was less stable than delphinidin- and cyanidin-3,5-diglucosides. There was a linear relationship between total monomeric anthocyanins and antioxidative capacity [116]. Consistently, liquid pomegranate peel extracts had acceptable thermal stability after sterilization and storage at low temperature [117]. Longer thermal treatment of juices (heating at 90°C for 5 h) resulted in total anthocyanin losses ranging from 76% to 87% of the initial anthocyanin levels. The anthocyanin stability was independent of the total phenolic content and of low and high molecular weight pomegranate matrix components (such as organic acids and sugars) [118]. Exposure to light during storage also affects loss of coactive compound [107, 117]. Good correlation of the anthocyanins with red colour was observed for all samples at elevated temperatures (70–90°C), but the visual appearance did not adequately reflect the quality and storage stability of pomegranate juices [118].

Ellagitannins seem to be the major antioxidants in pomegranate juices [106]. Commercial juices from whole pomegranates contained about 1500–1900 mg/L punicalagin while only traces of this compound were detected in self-made freshly squeezed juice from pomegranate arils. The ellagitannins in extracts from whole pomegranate are therefore derived from the peel [119]. Punicalagin concentrations ranged from 1100 to 2000 mg/kg dry matter of mesocarp and peel and from 4 to 565 mg/L in aril juices [119]. The punicalagin contents in the two pure juices analysed here are within or above this range (Table 2). For the whole pomegranate fruit extracts the punicalagin content of 95% relative to the total polyphenols and the low anthocyanin content of around 1% reflect the extraction from whole pomegranates (Table 2). The anthocyanins in the F4 preparation are derived from the additional elderberry as revealed by the individual elderberry anthocyanin components (Table 3).

Homogenates prepared from the whole fruit showed about a 20-fold higher antioxidant activity than did aril homogenates, which correlated significantly with the content of the four major hydrolyzable tannins (predominantly punicalagin) [120]. Likewise, when extracted with an ethanol-acetone extraction solvent, pomegranate peel showed greater antioxidant capacity than did pulp. This was consistent with the higher contents of total phenolics, flavonoids, and proanthocyanidins in the peel extract [121]. No correlation between antioxidant activity and level of anthocyanins was found [120]. When polyphenols purified from peel containing juice products were compared with those from peel-free juice, the radical scavenging effect was lower with the latter [122]. Juicing with peel made the juice bitter and astringent [122].

When 19 pomegranate food supplements were compared, only a limited number of pomegranate supplements were believably authentic. Product labels were inconsistent with polyphenol composition and antioxidant content. Thirteen samples contained disproportionately large amounts of ellagic acid and low or no detectable pomegranate tannins. Only six products had a tannin profile (punicalagin, punicalin, ellagitannins, and gallotannins) consistent with pomegranate. Natural pomegranate extract was the most representative of pomegranate fruit polyphenols with 99% total pomegranate polyphenol and the highest antioxidant capacity across all measures (Extract 1, lower part of Table 2. There were strong correlations between total polyphenols and antioxidant capacity in products that had polyphenol compositions consistent with a pomegranate source but not with products that contained large amounts of ellagic acid and little or no detectable pomegranate tannins. Thus, regulation of the market is required to assure consumers of the quality of pomegranate supplements [123, 124]. The content of saccharose and D-sorbit should be negligible, the glucose to fructose ratio should be below 1, the quantity of titratable acid (calculated as citric acid, pH 8.1) should vary between 1.9, and 45 g/L and malic acid should never be present. A comparative analysis of pomegranate juices and concentrates verified that many commercial products are mixed with sugar, colouring ingredients, and other fruit juices [112, 125]: these may or may not influence therapeutic effectiveness.

Processing conditions of the fruits, that is, coextraction of arils and peel and pressure, markedly affected the profiles and contents of phenolics in the pomegranate juices [108], underlining the necessity to optimise these features for obtaining products with well-defined and reproducible functional properties [119]. Heating plus refrigeration may help to reduce anthocyanin degradation in pasteurized pomegranate juice, avoiding a dramatic impact on its colour and preserving the beneficial effects [116]. Future research should identify the optimum coactive compound composition of a pomegranate preparation for the treatment of prostate cancer [120] with the primary outcome of 5-year survival. Declaration of the content of coactive constituents helps to identify quality products. However, direct evidence for bioequivalence between products can only come from well-planned clinical studies. Because of the complexity of the coactive pomegranate compounds, similar bioavailabilities of coactive compounds cannot provide indirect evidence for bioequivalence unless the clinical effectiveness for the leading polyphenol mixture has been confidently established [126]. This is because bioequivalence requires not only pharmaceutical similarity of components, but also their pharmacological and therapeutic equivalence.

Ellagic acid and its metabolites are found in human plasma after ingestion of pomegranate. Its antioxidant capacity was retained* ex vivo* [127]. An average ellagic acid serum concentration of 0.14 *μ*M/L was attained after consumption of a proprietary pomegranate extract and was associated with a putative anticancer effect [36]. Although similar ellagic acid serum concentrations were attained after taking extract or juice [23], another study showed lower concentrations of 0.06 *μ*M/L after drinking 180 mL of a pomegranate juice [128]. As long as we do not know which polyphenol (or polyphenols) is (or are) responsible for the putative anticancer effect, it is unwise to base dosing of pomegranate products on ellagic acid, because serum ellagic acid or its metabolites are the metabolites of various oligomeric polyphenols. Pharmacodynamic or* ex vivo/in vitro* tests are not surrogates for bioequivalence unless the results can be shown to correlate with therapeutic effectiveness [126].

In summary, there is evidence that pomegranate has a putative anticancerogenic effect in prostate cancer and can safely be used in high doses. But commercial pomegranate products vary greatly in their content of coactive ingredients. For reasons of transparency, consumers should know not only the photometric quantification of the polyphenols in the daily recommended dosage, but also the content of HPLC-analysed polyphenols. Only then can they choose a dose that has a chance of being effective in the treatment of cancer. The preparation of pomegranate end-products is affected by many determinants. Their declaration should be incorporated into the regulatory guidance and controlled before pomegranate products are allowed on the market.

## Figures and Tables

**Table 1:** Quality criteria considered in the 4 trials investigating pomegranate products.

	Clin cancer Res 2006; 12: 4018–26 Pantuck et al. [36]	Prostate cancer prostatic Dis 2013; 16: 50–5 Paller et al. [37]	J cancer 2013; 4: 597–605 Stenner-Liewen et al. [40]	Cancer prev Res (Phila) 2013; 6: 1120–7 Freedland et al. [38]
	*N* = 46	*N* = 92	*N* = 97	*N* = 69
SM	Juice POM wonderful	extract POMx	Pomegranate blend	Extract POMx
Dose	240 mL/day	1000 mg versus 2000 mg/day	500 mL/day	2 × 1000 mg/day
pa	570 mg/day	Not stated	700 mg/day^*^	1200 mg/day
cai	Not stated^*^	400 mg versus 800 mg/day	40 mg/day	Not stated^*^
	Open, uncontrolled	Low dose (45), high dose (47)	Placebo (48), control (49)	Placebo (36), control (33)
D	13 months	Up to 18 months	4 weeks	4 weeks
R	Lengthening of PSA doubling time	No difference between groups lengthening of PSA doubling time	No difference between groups	No difference between groups
A	Prostate cancer	Prostate cancer	Prostate cancer	Prostate cancer requiring Radical prostatectomy
B	Not applicable	Not stated	Yes	Yes
C	Not applicable	Not stated	Yes	Yes
E	Not applicable	Not stated	Yes	Yes
F	Not applicable	Yes	Yes	Yes
G	Not applicable	Yes	Yes	Yes
H	Yes	Yes	Yes	Yes
I	Yes	Yes	Yes	Not stated
J	Yes	Yes	Yes	Yes (none)
**K**	Yes	Yes	Yes	Yes
L	No	Yes	No	No
N	No	No	No	No
O	Yes	Yes	No	No

TS	6	9	10	9

	^*^according to Paller the same as in extract POMx	^*^from other source see Hong et al., 2008 [14]	^*^see Chrubasik-Hausmann et al. 2014a [41]	^*^no details given on request

SM study medication, pa photometrically assessed, cai coactive ingredients/day (HPLC) D duration of treatment, R result.

Quality criteria A: eligibility criteria specified, B: randomization appropriate, C: treatment allocation concealed, E: similarity at baseline, F: outcome measures and control interventions explicitly described, G: cointerventions comparable, H: outcome measures relevant, I: adverse events and J drop-outs fully described, K: sample size based on a priori power calculation, L: intention-to-treat analysis, N: point estimates and measures of variability presented for the primary outcome measure, and O: appropriate timing giving a total score (TS) of 13.

**Table 2:** Content of coactive ingredients in various pomegranate preparations (density of liquid products 1.3).

Preparation	Total polyphenols		Total anthocyanins	Punicalagin A + B	Ellagic acid	^***^Sum of A, P, and EA	
	Declared	Measured					Daily Dose
Mother juice 5174-13	n.i.	2654 mg/L^*^	34.47 mg/L	271 mg/L	81.5 mg/L	387 mg/L	39 mg/100 mL
Mother juice L3074	3840 mg/L^**^	2188 mg/L^*^	9.45 mg/L	948 mg/L	47.4 mg/L	1005 mg/L	101 mg/100 mL
POM wonderful juice	n.i.	2670 mg/L^*^	60.7 mg/L	310 mg/L	134 mg/L	505 mg/L	121/240 mL
POM Wonderful Concentrate	n.i.	18900 mg/L^*^	1.1 mg/L	1400 mg/kg	146 mg/kg	1547 mg/L	77 mg/50 mL
F4 concentrate	71515 mg/L^*^	73944 mg/L^*^	179.4 mg/L	29900 mg/L	1378 mg/L	31457 mg/L	315 mg/10 mL
POMx-capsules	n.i.	613000 mg/kg^*^	n.d.	103000 mg/kg	28700 mg/kg	131700 mg/kg	132 mg/1000 mg
Ultra Granatapfel forte capsules	n.i.	843000 mg/kg^*^	17.3 mg/kg	45900 mg/kg	13900 mg/kg	59800 mg/kg	30 mg/500 mg
Extract 20651	59000 mg/kg^**^	189900 mg/kg^*^	241 mg/kg	38400 mg/kg	1610 mg/kg	40251 mg/kg	40 mg/1000mg
GranaProstan capsules	460000 mg/kg^*^	394000 mg/kg^*^	112 mg/kg	74000 mg/kg	69900 mg/kg	144012 mg/kg	142 mg/1000 mg

	Reference						
POM Wonderful juice (mg/L)	McCutcheon et al., 2008 [102]		n.i.	1740^&^	140^&^	1860^&^	94 mg/240 mL
POM Wonderful Concentrate (mg/L)	http://www.google.ca/patents/US7727563 ^ &^		384^§^	1561^§^	121^§^	2066^§^	103 mg/50 mL
Extract 1 (mg/1000 g)	Madrigal-Carballo et al., 2009 [123]		n.i.	177000^§^	33000^§^	n.i.	201 mg/1000 mg
Pomella (mg/kg)	Patel et al., 2008 [48]		none^§^	300000^§^	21500^§^	321500	193 mg/600 mg
POMx wonderful (mg/kg)	Hong et al., 2008 [14]		n.i.	370000^§^	30000^§^	n.i.	400 mg/1000 mg

^*^total polyphenols calculated as gallic acid equivalents (Folin-Ciocalteau, photometric assessment).

^**^total polyphenols calculated as pyrogallol (PhEur 2.8.14, photometric assessment).

^***^sum of anthocyanins (A), punicalagin (P), and ellagic acid (EA) assessed by HPLC; DD in the suggested daily dose.

n.d.: not detectable.

n.i. no information.

^§^according to the manufacturers, ^&^according to the POM wonderful monography.

**Table 3:** Content of individual anthocyanins expressed as cyanidin-3-glucoside equivalents (mg/L at 500 nm) in the pomgranate preparations investigated.

	Delphinidin-3,5-diglucoside	Cyanidin-3,5-diglucoside	Delphinidin-3-glucoside + pelargonidin-3,5-glucoside + cyanidin-rutinoside	Cynanidin-3-glucoside	Delphinidin-pentoside	Pelargonidin-3-glucoside	Cyanidin-pentoside
Mother juice 5174-13 (mg/L)	5.63	15.0	4.07	8.79	n.d.	0.77	0.21
Mother juice L3074 (mg/L)	2.14	5.11	0.79	1.32	n.d.	0.11	n.d.
POM wonderful							
Juice (mg/L)	7.3	13.2	7.0	17.2	1.7	1.3	n.d.
Concentrate (mg/L)	n.d.	n.d.	n.d.	1.1	n.d.	n.d.	n.d.
F4 concentrate (mg/L)	n.d.	20.3^*^	n.d.	59.9^**^	n.d.	57.4	n.d.
POMx-capsules (mg/kg)	n.d.	n.d.	n.d.	n.d.	n.d.	n.d.	n.d.
Ultra Granatapfel forte							
Capsules (mg/kg)	12.3	n.d.	4.9	n.d.	n.d.	n.d.	n.d.
Extract 20651 (mg/kg)	20.3	63.4	36.6	88.5	18.0	14.2	n.d.
GranaProstan capsules (mg/kg)	n.d.	n.d.	32.0	13.4	29.0	n.d.	0.9
POM wonderful concentrate^&^	n.i.	n.i.	n.i.	1.1	n.i.	n.i.	n.i.

^*^Sum of cyanidin-3,5-diglucoside and cyanidin-3-sambubioside-5-glucoside of elderberry.

^**^Sum cyanidin-3-glucoside and cyanidin-3-sambusoside of elderberry.

n.i. no information.

^&^adopted from http://www.google.ca/patents/US7727563.
